# Supplementary material for: The allosteric modulation of complement C5 by knob domain peptides
Source: eLife. 2021 Feb 11;10:e63586. doi: 10.7554/eLife.63586 (PMC7972453; doi:10.7554/eLife.63586)

**Supplementary File**

**The allosteric modulation of Complement C5 by knob domain peptides**

Alex Macpherson^1,2^*, Maisem Laabei^2^, Zainab Ahdash^1^, Melissa Graewert^3,4^, James R. Birtley^1^, Monika-Sarah E.D. Schulze^1^, Susan Crennell^2^, Sarah A. Robinson^4^, Ben Holmes^1^, Vladas Oleinikovas^1^, Per H. Nilsson^5,6^, James Snowden^1^, Victoria Ellis^1^, Tom Eirik Mollnes^6,7,8^, Charlotte M. Deane^4^, Dmitri Svergun^3^, Alastair D.G. Lawson^1^ and Jean van den Elsen^2,9^*

^1^ UCB, Slough, UK. SL1 3WE;

^2^ Department of Biology and Biochemistry, University of Bath, Bath, UK. BA2 7AX;

^3^ European Molecular Biology Laboratory, Hamburg Unit, 22607 Hamburg, Germany;

^4^ Department of Statistics, University of Oxford, Oxford, UK;

^5^ Department of Chemistry and Biomedicine, Linnaeus University, 391 82 Kalmar, Sweden;

^6^ Department of Immunology, Oslo University Hospital, University of Oslo, Oslo, Norway;

^7^ Research Laboratory, Bodø Hospital, K.G. Jebsen TREC, University of Tromsø, Tromsø, Norway;

^8^ Centre of Molecular Inflammation Research, Norwegian University of Science and Technology, Trondheim, Norway;

^9^ Centre for Therapeutic Innovation, University of Bath, Bath, UK. BA2 7AX

*email [alex.macpherson@ucb.com](mailto:alex.macpherson@ucb.com) and [bssjmhve@bath.ac.uk](mailto:bssjmhve@bath.ac.uk)

**Section 1. Functional analyses**

**Table 1.1. Classical pathway C5b deposition ELISA.**

Data from *n=3*, unless specified.

| **Construct** | **Geomean IC50 (nM)** | **Range (nM)** | **Average Emax (%)** | **Range (%)** |
| --- | --- | --- | --- | --- |
| **K8^a^** | 9.3 | 3.0-24.5 | 68.2 | 59.5 – 79.56 |
| **K57** | 3.6 | 2.4 – 7.3 | 100.6 | 99.1 – 101.7 |
| **K92** | ND* | ND* | ND* | ND* |
| **K149** | ND* | ND* | ND* | ND* |

*ND = Not detected.

^a^ Data are an average from *n=6*

**Table 1.2. Alternative pathway C5b deposition ELISA.**

Data from *n=3*, unless specified.

| **Construct** | **Geomean IC50 (nM)** | **Range (nM)** | **Average Emax (%)** | **Range (%)** |
| --- | --- | --- | --- | --- |
| **K8 ^a^** | 29.2 | 25.4 – 34.2 | 91.8 | 91.4 – 92.3 |
| **K57** | 30.4 | 22.7 – 52.1 | 110.3 | 97.9 – 134.0 |
| **K92^b^** | 32.4 | 31.5 - 33.1 | 62.14 | 58.0 - 67.7 |
| **K149** | ND* | ND* | ND* | ND* |

*ND = Not detected.

^a^ Data are an average from *n=5*

^b^ Data are an average from *n=4*

**Table 1.3. Inhibition of classical pathway mediated C5a release.**

Data from *n=3* independent titrations, unless otherwise stated

| **Construct** | **Geomean IC50 (nM)** | **Range (nM)** | **Average Emax (%)** | **Range (%)** |
| --- | --- | --- | --- | --- |
| **K8 ^a^** | 9.9 | 3.9 – 18.7 | 57.7 | 51.5 – 73.2 |
| **K57** | 4.0 | 2.8 - 5.6 | 98.7 | 95.9 - 100.6 |
| **K92** | ND* | ND* | ND* | ND* |
| **K149** | ND* | ND* | ND* | ND* |

*ND = Not detected.

^a^ Data are an average from *n=5*

**Table 1.4. Inhibition of alternative pathway mediated C5a release.**

Data from *n=3*, unless otherwise stated

| **Construct** | **Geomean IC50 (nM)** | **Range (nM)** | **Average Emax (%)** | **Range (%)** |
| --- | --- | --- | --- | --- |
| **K8^a^** | 150.2 | 49.3 – 85.86 | 71.8 | 49.3 – 85.9 |
| **K57** | 26.6 | 25.0-27.7 | 97.8 | 95.1 - 101.3 |
| **K92** | 43.3 | 39.9 – 45.3 | 43.3 | 39.9 – 45.2 |
| **K149** | ND* | ND* | ND* | ND* |

*ND = Not detected.

^a^ Data are an average from *n=4*

**Table 1.5. Inhibition of classical pathway mediated C9 deposition.**

Data from *n=3*

| **Construct** | **Geomean IC_50_ (nM)** | **Range (nM)** | **Average Emax (%)** | **Range (%)** |
| --- | --- | --- | --- | --- |
| **K8^#^** | - | - | 63.4 | - |
| **K57** | 1.9 | 1.3-2.5 | 97.9 | 92.4 - 97 .9 |
| **K92** | ND* | ND* | ND* | ND* |
| **K149** | ND* | ND* | ND* | ND* |

*ND = Not detected.

^#^ Hill slopes < 0.5 IC_50_ values not reported.

**Table 1.6. Inhibition of alternative pathway mediated C9 deposition.**

Data from *n=3*, unless otherwise stated.

| **Construct** | **Geomean IC_50_ (nM)** | **Range (nM)** | **Average Emax (%)** | **Range (%)** |
| --- | --- | --- | --- | --- |
| **K8** | 232.4 | 131.3 – 521.5 | 73.3 | 61.2 – 90.74 |
| **K57** | 24.5 | 20.6 - 27.6 | 100.7 | 100.1 - 100.9 |
| **K92** | 791.9 | 759.8 - 846.7 | 44.2 | 40.9 – 45.9 |
| **K149** | ND* | ND* | ND* | ND* |

*ND = Not detected.

**Table 1.7. Inhibition of classical pathway haemolysis.**

Data from *n=3*

| **Construct** | **Geomean IC_50_ (nM)** | **Range (nM)** | **Average Emax (%)** | **Range (%)** |
| --- | --- | --- | --- | --- |
| **K8^#^** | - | - | 95.6 | 91.7 – 99.7 |
| **K57** | 5.3 | 4.9 – 5.7 | 99.7 | 99.2 –100.1 |
| **K92** | ND* | ND* | ND* | ND* |

*ND = Not detected.

^#^ curves not well described by a 4-PL fit, IC_50_ values not reported.

**Table 1.8. Inhibition of alternative pathway haemolysis.**

Data from *n=3*

| **Construct** | **Geomean IC_50_ (nM)** | **Range (nM)** | **Average Emax (%)** | **Range (%)** |
| --- | --- | --- | --- | --- |
| **K8** | ND* | ND* | ND* | ND* |
| **K57** | 402.7 | 218 – 660 | 102.2 | 90.9 – 109.6 |
| **K92** | 16.9 | 11.41 – 21.8 | 31.4 | 24.1 – 45.3 |

*ND = Not detected.

**Section 2. Structural analysis**

**Table 2.1. Data collection and refinement statistics (molecular replacement)**

| **Data collection** | **C5-K8** | **C5-K92** |
| --- | --- | --- |
| Space group | P 21 21 21 | C 1 2 1 |
| Cell dimensions |  |  |
| *a*, *b*, *c* (Å) | 81.56, 161.69, 187.38 | 204.27, 104.29, 154.88 |
| α, β, γ (°) | 90, 90, 90 | 90, 124.89, 90 |
| Resolution (Å) | 81.07-2.3 (2.382-2.3)[^a^](https://www.ncbi.nlm.nih.gov/pmc/articles/PMC5383516/table/T1/#TFN1) | 83.77-2.75 (2.848-2.75) |
| *R*_merge_ | 0.043 (0.361) | 0.018 (0.204) |
| *I* / σ*I* | 13.17 (2.07) | 28.79 (4.38) |
| CC_1/2_ | 0.99 (0.67) | 0.99 (0.92) |
| Completeness (%) | 0.99 (0.99) | 0.99 (0.99) |
| Redundancy | 2.0 (2.0) | 2.0 (2.0) |
| **Refinement** |  |  |
| Resolution (Å) | 81.56-2.3 | 83.77-2.75 |
| No. reflections | 110479 (10914) | 69381 (6877) |
| *R*_work_ / *R*_free_ | 0.202/0.234 | 0.219/0.253 |
| No. atoms (non-H) | 12100 | 13095 |
| Protein | 11618 | 13024 |
| Ligand/ion | 80 | 59 |
| Water | 402 | 12 |
| *B* factors |  |  |
| Protein | 65.4 | 100.5 |
| Ligand/ion | 87.9 | 122.7 |
| Water | 51.9 | 65.3 |
| r.m.s. deviations |  |  |
| Bond lengths (Å) | 0.003 | 0.002 |
| Bond angles (°)  Ramachandran plot (%)  Favoured  Allowed  Outliers  Clash score | 0.62  97.2  2.8  0  3.3 | 0.58  95.2  4.8  0  6.7 |

^a^Values in parentheses are for highest-resolution shell.

**Table 2.2. Hydrogen bond interactions between K8 and C5**

Hydrogen bonding interactions as defined by the PDBePiSA macromolecular interfaces tool.

| **K8** | | **C5** | |
| --- | --- | --- | --- |
| *Hydrogen bonds (18)* | | | |
| A14 | O | N | L1379 |
| G16 | N | O | L1379 |
|  | O | N | I1381 |
| S18 | N | OD2 | D1382 |
|  | OG | NZ | K1380 |
| R23 | NH1 | O | E1373 |
|  |  | O | S1371 |
|  | NH2 | O | E1373 |
| D25 | OD2 | OH | Y1378 |
|  | OD2 | NZ | K1409 |
| R32 | NH1 | OD2 | D1471 |
|  | NH2 | OD1 |  |
| H36 | NE2 | OD1 | D1382 |
| G43 | N | OG | S1407 |
| N44 | N | O | S1469 |
|  | ND2 | O | S1470 |
| R45 | NH1 | O | S1411 |
|  | NH2 | O | E1414 |

**Table 2.3. Ionic interactions between K8 and C5.**

Ionic interactions as defined by the PDBePiSA macromolecular interfaces tool.

| **K8** | | **C5** | |
| --- | --- | --- | --- |
| *Ionic interactions (5)* | | | |
| D25 | OD2 | NZ | K1409 |
| R32 | NH1 | OD2 | D1471 |
|  | NH2 | OD1/OD2 |  |
| H36 | NE2 | OD1 | D1382 |

**Table 2.4. Disulphide mapping of the K92 peptide.**

| Intensities and resulting % (by total intensity) for the various peptides linked by a single disulphide bond as identified by Biopharma Finder. | | |
| --- | --- | --- |
| **Cysteine Pairing** | **Intensity^a^** | **% (by total intensity)** |
| Cys23 to Cys9 | 150564035 | 62.71 |
| Cys18 to Cys2 | 85987873 | 35.81 |
| Cys23 to Cys2 | 2796033 | 1.16 |
| Cys23 to Cys23 | 527299 | 0.22 |
| Cys18 to Cys9 | 166812 | 0.07 |
| Cys23 to Cys18 | 17112 | 0.01 |
| Cys2 to Cys2 | 15354 | 0.01 |
| Cys18 to Cys18 | 11924 | 0.00 |
| Cys2 to Cys9 | 5193 | 0.00 |
| ^a^ Sum of intensities of all peptides (containing a single disulphide bond) identified by Biopharma Finder as containing the same paired cysteines. | | |

**Table 2.5. Hydrogen bond interactions between K92 and C5.**

Hydrogen bonding interactions as defined by the PDBePiSA macromolecular interfaces tool.

| **K92** | | **C5** | |
| --- | --- | --- | --- |
| ***Hydrogen bonds (8)*** | | | |
| G22 | N | OD1 | N81 |
| H25 | NE2 | O | A77 |
| F26 | N | O | F512 |
| I13 | O | ND2 | N533 |
| C23 | O | ND2 | N38 |
|  | O | OG | S82 |
|  | N | OG | S82 |
| G24 | O | N | F512 |

**Table 2.6.** **Validation of molecular interactions by peptide mutagenesis analysis.**

Summary of SPR multi-cycle kinetics

| **knob domain** | **mean k_on_ (m s^-1^)** | **k_on_ SE** | **mean k_off_** | **k_off_ SD** | **mean K_D_ (M)** | **mean stoich. ratio** |
| --- | --- | --- | --- | --- | --- | --- |
| K92 | 2.83E+05 | 3.79E+04 | 1.01E-04 | 6.32E-05 | 4.11E-10 | 0.40 |
| K92 W21A | 1.74E+04 | 7.10E+02 | 8.48E-03 | 1.13E-03 | 4.97E-07 | 0.62 |
| K92 F26A | 1.84E+05 | 2.58E+04 | 3.28E-03 | 7.12E-04 | 1.88E-08 | 0.65 |
| K8 | 7.05E+04 | 8.23E+02 | 3.85E-04 | 3.25E-05 | 5.46E-09 | 0.25 |
| K8 R22A | 5.40E+04 | 1.65E+03 | 5.99E-04 | 6.31E-05 | 1.12E-08 | 0.27 |
| K8 R31A | 4.62E+02 | 9.45E+01 | 1.52E-03 | 2.86E-04 | 3.91E-06 | 0.75 |

*n=1*

|  | **k_on_** | **k_off_** | **K_D_ (M)** | **stoich. ratio** |
| --- | --- | --- | --- | --- |
| K92 | 1.86E+05 | 8.17E-05 | 4.40E-10 | 0.54 |
| K92 W21A | 1.91E+04 | 8.35E-03 | 4.37E-07 | 0.60 |
| K92 F26A | 2.71E+05 | 4.03E-03 | 1.49E-08 | 0.67 |
| K8 | 7.10E+04 | 3.72E-04 | 5.24E-09 | 0.28 |
| K8 R22A | 5.92E+04 | 5.65E-04 | 9.54E-09 | 0.29 |
| K8 R31A | 7.86E+02 | 1.82E-03 | 2.32E-06 | 0.36 |

*n=2*

|  | **k_on_** | **k_off_** | **K_D_ (M)** | **stoich. ratio** |
| --- | --- | --- | --- | --- |
| K92 | 4.08E+05 | 5.00E-05 | 1.23E-10 | 0.33 |
| K92 W21A | 1.81E+04 | 7.42E-03 | 4.11E-07 | 0.57 |
| K92 F26A | 1.21E+05 | 2.61E-03 | 2.15E-08 | 0.63 |
| K8 | 7.26E+04 | 4.22E-04 | 5.81E-09 | 0.23 |
| K8 R22A | 5.35E+04 | 6.72E-04 | 1.26E-08 | 0.24 |
| K8 R31A | 3.36E+02 | 1.25E-03 | 3.72E-06 | 0.71 |

*n=3*

|  | **k_on_** | **k_off_** | **K_D_ (M)** | **stoich. ratio** |
| --- | --- | --- | --- | --- |
| K92 | 2.56E+05 | 1.72E-04 | 6.72E-10 | 0.32 |
| K92 W21A | 1.50E+04 | 9.66E-03 | 6.44E-07 | 0.68 |
| K92 F26A | 1.61E+05 | 3.21E-03 | 1.99E-08 | 0.65 |
| K8 | 6.78E+04 | 3.61E-04 | 5.32E-09 | 0.24 |
| K8 R22A | 4.94E+04 | 5.61E-04 | 1.13E-08 | 0.27 |
| K8 R31A | 2.63E+02 | 1.49E-03 | 5.68E-06 | 1.18 |

**Table 2.7. Individual, total and average hydrogen bond persistence in a binding pose metadynamics simulation of the K8-C5 complex.**

| **HBond K8** | **HBond C5** | **Persistence score** |
| --- | --- | --- |
| 14:O | 1379:H | 1.000 |
| 16:H | 1379:O | 0.018 |
| 16O | 1381:H | 1.000 |
| 18:H | 1382:OD1(OD2) | 1.000 |
| 23:HH12(HH11,HH21,HH22) | 1373:O | 0.355 |
| 23:HH22(HH11,HH12,HH21) | 1373:O | 0.355 |
| 25:OD2(OD1) | 1378:HH | 0.964 |
| 32:HH12(HH11,HH21,HH22) | 1471:OD2(OD1) | 0.891 |
| 32:HH22(HH11,HH12,HH21) | 1471:OD2(OD1) | 0.891 |
| 43:H | 1407:OG | 0.609 |
| 44:HD22(HD21) | 1470:O | 0.900 |
| Total: | | 7.98 |
| Average: | | 0.726 |

**Table 2.8.** **Individual, total and average hydrogen bond persistence in a binding pose metadynamics simulation of the K92-C5 complex.**

| **HBond K92** | **HBond C5** | **Persistence score** |
| --- | --- | --- |
| 13:O | 533:HD21(HD22) | 0.909 |
| 22:H | 81:OD1 | 0.209 |
| 23:O | 38:HD22(HD21) | 0.536 |
| 24:O | 512:H | 0.536 |
| 25:HE2 | 80:O | 0.545 |
| Total: | | 2.74 |
| Average: | | 0.547 |

**Section 3. Solution structure analysis**

**Table 3.1. SAXS summary data.**

| **Data collection parameters** | **C5** | **C5-K8** | | **C5-K57** | | **C5-K92** | **CK-149:** |
| --- | --- | --- | --- | --- | --- | --- | --- |
| Instrument | EMBL P12 (PETRA III, DESY, Hamburg) | | | | | | |
| Beam geometry (mm^2^) | 0.2×0.12 | | | | | | |
| Wavelength (nm) | 12.4 | | | | | | |
| *s* range (nm^-1^) | 0.03-7.1 | | | | | | |
| Temperature (K) | 293 | | | | | | |
| Concentration range (ml) | 5.96 | | | | | | |
| **SEC parameters** |  | | | | | | |
| Column | Superdex 200 Increase 5/150 column | | | | | | |
| Buffer | 20mM Tris pH 7.35, 75mM NaCl, and 3% glycerol | | | | | | |
| Flow rate, duration | 0.35 ml/min; 15 min (900 frames) | | | | | | |
| **Structural parameters** |  | |  | |  | | |
| *R*g (nm) (from *P*(*r*)) | 4.8 ± 0.1 | 5.4 ± 0.1 | | 5.2 ± 0.1 | | 5.0 ± 0.1 | 4.9 ± 0.1 |
| *R*g (nm) (from Guinier plot) | 4.8 ± 0.1 | 5.4 ± 0.1 | | 5.3 ± 0.1 | | 5.1 ± 0.1 | 5.0 ± 0.1 |
| *D*max (nm) | 17.6 ± 0.5 | 18.3 ± 10 | | 19 ± 0.5 | | 19 ± 0.5 | 18.5 ± 0.5 |
| Porod volume estimate, V_p_ (nm3) | 390 ± 10 | 450 ± 10 | | 410 ± 10 | | 410 ± 10 | 410 ± 10 |
| **Molecular weight determination (kDa)** |  |  | |  | |  |  |
| From volume of correlation, VC | 210 ± 20 | 240 ± 25 | | 230 ± 25 | | 220 ± 22 | 220 ± 22 |
| From *MALLS* | 188 ± 9 | 200 ± 10 | | 199 ± 10 | | 197 ± 10 | 195 ± 10 |
| Calculated monomeric *MW* from sequence | 186 | 192 | | 190 | | 191.5 | 190 |
| **Software employed** |  |  | |  | |  |  |
| Primary data reduction | SASFLOW | | | | | | |
| Data processing | CHROMIXS/PRIMUS | | | | | | |
| Computation of model intensities | CRYSOL | | | | | | |
| normal mode analysis | SREFLEX | | | | | | |

**Table 3.2. ΔHDX summary data.**

| Data set | **ΔHDX = (C5 + K8) – (C5)** | **ΔHDX = (C5 + K57) – (C5)** | **ΔHDX = (C5 + K92) – (C5)** |
| --- | --- | --- | --- |
| Protein buffer conditions | PBS, pH 7.2 | | |
| Deuterium time course analysed | 30, 120, 900 and 3600 sec | | |
| Number of peptides | 248 | 188 | 156 |
| Sequence coverage | 73.3 % | 67.0 % | 61.3 % |
| Redundancy | 2.43 | 2.10 | 2.43 |
| Replicates | Triplicates | | |
| Average standard deviation | 0.06 | 0.08 | 0.09 |
| Significant differences at 1 hour ΔHDX* | CI 98% = 0± 24 Da | CI 98% = 0± 33 Da | CI 98% = 0± 39 Da |

* To compare significant differences, a T-test with α=0.02 was used. Only peptides which satisfied a ΔHDX confidence interval of 98 % were considered significant.

**Section 4. Additional functional analyses**

**Table 4.1. SPR single-cycle kinetics of knob domains binding to human C5b.**

summary of *n=3*

|  | **Chi² (RU²)** | **mean k_on_ (1/Ms)** | **mean k_off_ (1/s)** | **mean K_D_ (M)** | **Mean stoichiometric ratio** | **comment** |
| --- | --- | --- | --- | --- | --- | --- |
| K8 | - | - | - | - | 0.1 | Non-binding |
| K92 | 0.04 | 1.14E+05 | 6.20E-05 | 5.48E-10 | 0.4 |  |
| K57 | 0.07 | 2.51E+05 | 3.54E-04 | 1.41E-09 | 0.4 |  |
| K149 | 0.03 | 7.49E+05 | 3.76E-03 | 5.02E-09 | 0.4 |  |

*n=1*

|  | **Chi² (RU²)** | **k_on_ (1/Ms)** | **k_off_ (1/s)** | **K_D_ (M)** | **Rmax (RU)** | **Stoichiometric ratio** | **comment** |
| --- | --- | --- | --- | --- | --- | --- | --- |
| K8 | - | - | - | - | 2.1 | 0.1 | Non-binding |
| K92 | 0.06 | 1.11E+05 | 5.20E-05 | 4.67E-10 | 5.4 | 0.4 |  |
| K57 | 0.05 | 2.73E+05 | 3.75E-04 | 1.37E-09 | 6.2 | 0.4 |  |
| K149 | 0.03 | 7.56E+05 | 3.65E-03 | 4.83E-09 | 2.8 | 0.3 |  |

*n=2*

|  | **Chi² (RU²)** | **k_on_ (1/Ms)** | **k_off_ (1/s)** | **K_D_ (M)** | **Rmax (RU)** | **Stoichiometric ratio** | **comment** |
| --- | --- | --- | --- | --- | --- | --- | --- |
| K8 | - | - | - | - | 2.4 | 0.1 | Non-binding |
| K92 | 0.03 | 1.10E+05 | 7.68E-05 | 6.99E-10 | 5.2 | 0.4 |  |
| K57 | 0.08 | 2.40E+05 | 3.46E-04 | 1.44E-09 | 8.2 | 0.4 |  |
| k149 | 0.03 | 7.80E+05 | 3.99E-03 | 5.11E-09 | 5.1 | 0.4 |  |

*n=3*

|  | **Chi² (RU²)** | **k_on_ (1/Ms)** | **k_off_ (1/s)** | **K_D_ (M)** | **Rmax (RU)** | **Stoichiometric ratio** | **comment** |
| --- | --- | --- | --- | --- | --- | --- | --- |
| K8 | - | - | - | - | 2.6 | 0.2 | Non-binding |
| K92 | 0.03 | 1.20E+05 | 5.73E-05 | 4.78E-10 | 4.7 | 0.3 |  |
| K57 | 0.08 | 2.38E+05 | 3.42E-04 | 1.43E-09 | 7.9 | 0.4 |  |
| K149 | 0.03 | 7.12E+05 | 3.65E-03 | 5.13E-09 | 5.2 | 0.4 |  |

Example sensorgrams and curve fits (1:1 binding model) are shown with summary kinetics from *n=3* experiments.


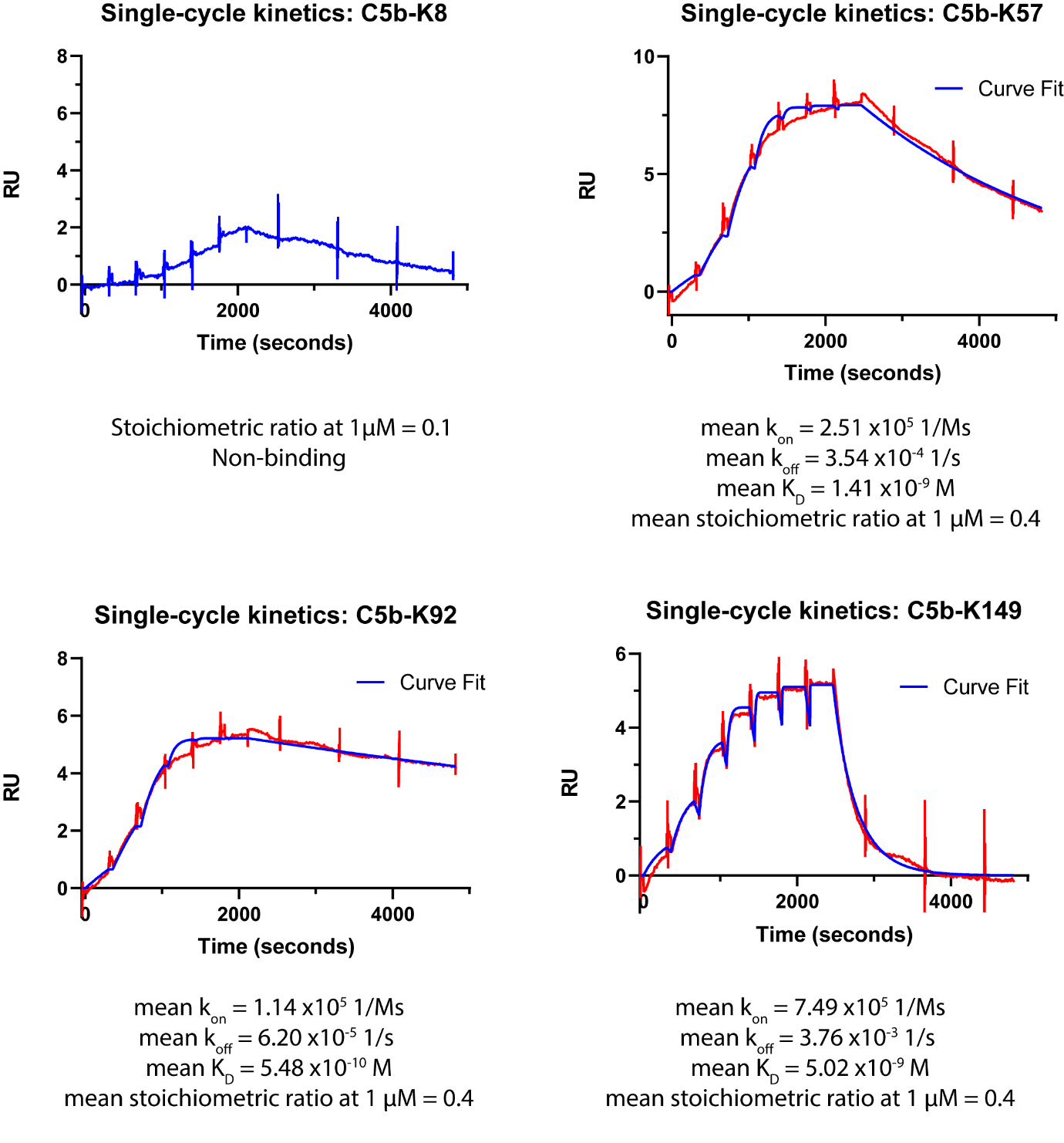


**Table 4.2. SPR single-cycle kinetics of knob domains binding to human C5b-6.**

summary of *n=4*

|  | **mean k_on_ (1/Ms)** | **mean k_off_ (1/s)** | **mean K_D_ (M)** | **Mean stoichiometric ratio** |
| --- | --- | --- | --- | --- |
| K8 | 1.25E+04 | 2.95E-04 | 2.43E-08 | 0.4 |
| K57 | 1.50E+05 | 5.93E-04 | 3.91E-09 | 0.8 |
| K92 | 1.36E+04 | 8.12E-05 | 6.18E-09 | 0.5 |
| K149 | 1.24E+06 | 4.88E-03 | 3.98E-09 | 0.7 |

*n=1*

|  | **Chi² (RU²)** | **k_on_ (1/Ms)** | **k_off_ (1/s)** | **K_D_ (M)** | **Rmax (RU)** | **Stoichiometric ratio** |
| --- | --- | --- | --- | --- | --- | --- |
| K8 | 4.57E-02 | 1.01E+04 | 2.82E-04 | 2.78E-08 | 6.1 | 0.3 |
| K57 | 5.21E-02 | 1.48E+05 | 5.72E-04 | 3.87E-09 | 9.7 | 0.7 |
| K92 | 8.49E-02 | 1.16E+04 | 9.65E-05 | 8.33E-09 | 9.5 | 0.5 |
| K149 | 2.75E-01 | 1.33E+06 | 4.78E-03 | 3.58E-09 | 7.2 | 0.7 |

*n=2*

|  | **Chi² (RU²)** | **k_on_ (1/Ms)** | **k_off_ (1/s)** | **K_D_ (M)** | **Rmax (RU)** | **Stoichiometric ratio** |
| --- | --- | --- | --- | --- | --- | --- |
| K8 | 1.41E-02 | 1.21E+04 | 3.84E-04 | 3.17E-08 | 5.4 | 0.3 |
| K57 | 2.60E-02 | 1.41E+05 | 4.81E-04 | 3.40E-09 | 11.7 | 0.7 |
| K92 | 1.91E-02 | 1.27E+04 | 9.86E-05 | 7.77E-09 | 9.8 | 0.5 |
| K149 | 1.80E-01 | 1.30E+06 | 4.39E-03 | 3.37E-09 | 6.9 | 0.6 |

*n=3*

|  | **Chi² (RU²)** | **k_on_ (1/Ms)** | **k_off_ (1/s)** | **K_D_ (M)** | **Rmax (RU)** | **Stoichiometric ratio** |
| --- | --- | --- | --- | --- | --- | --- |
| K8 | 1.94E-01 | 1.21E+04 | 2.40E-04 | 1.98E-08 | 11.8 | 0.5 |
| K57 | 1.51E-01 | 1.65E+05 | 8.14E-04 | 4.92E-09 | 14.6 | 0.8 |
| K92 | 5.49E-02 | 1.54E+04 | 6.78E-05 | 4.42E-09 | 14.1 | 0.6 |
| K149 | 2.85E-01 | 1.18E+06 | 5.37E-03 | 4.53E-09 | 10.3 | 0.8 |

*n=4*

|  | **Chi² (RU²)** | **k_on_ (1/Ms)** | **k_off_ (1/s)** | **K_D_ (M)** | **Rmax (RU)** | **Stoichiometric ratio** |
| --- | --- | --- | --- | --- | --- | --- |
| K8 | 7.38E-02 | 1.56E+04 | 2.77E-04 | 1.77E-08 | 7.6 | 0.3 |
| K57 | 1.38E-01 | 1.47E+05 | 5.05E-04 | 3.43E-09 | 16.1 | 0.9 |
| K92 | 5.62E-02 | 1.48E+04 | 6.19E-05 | 4.19E-09 | 14.3 | 0.6 |
| K149 | 4.26E-02 | 1.12E+06 | 4.97E-03 | 4.42E-09 | 10.8 | 0.8 |

Example sensorgrams and curve fits (1:1 binding model) are shown with summary kinetics from *n=3* experiments.


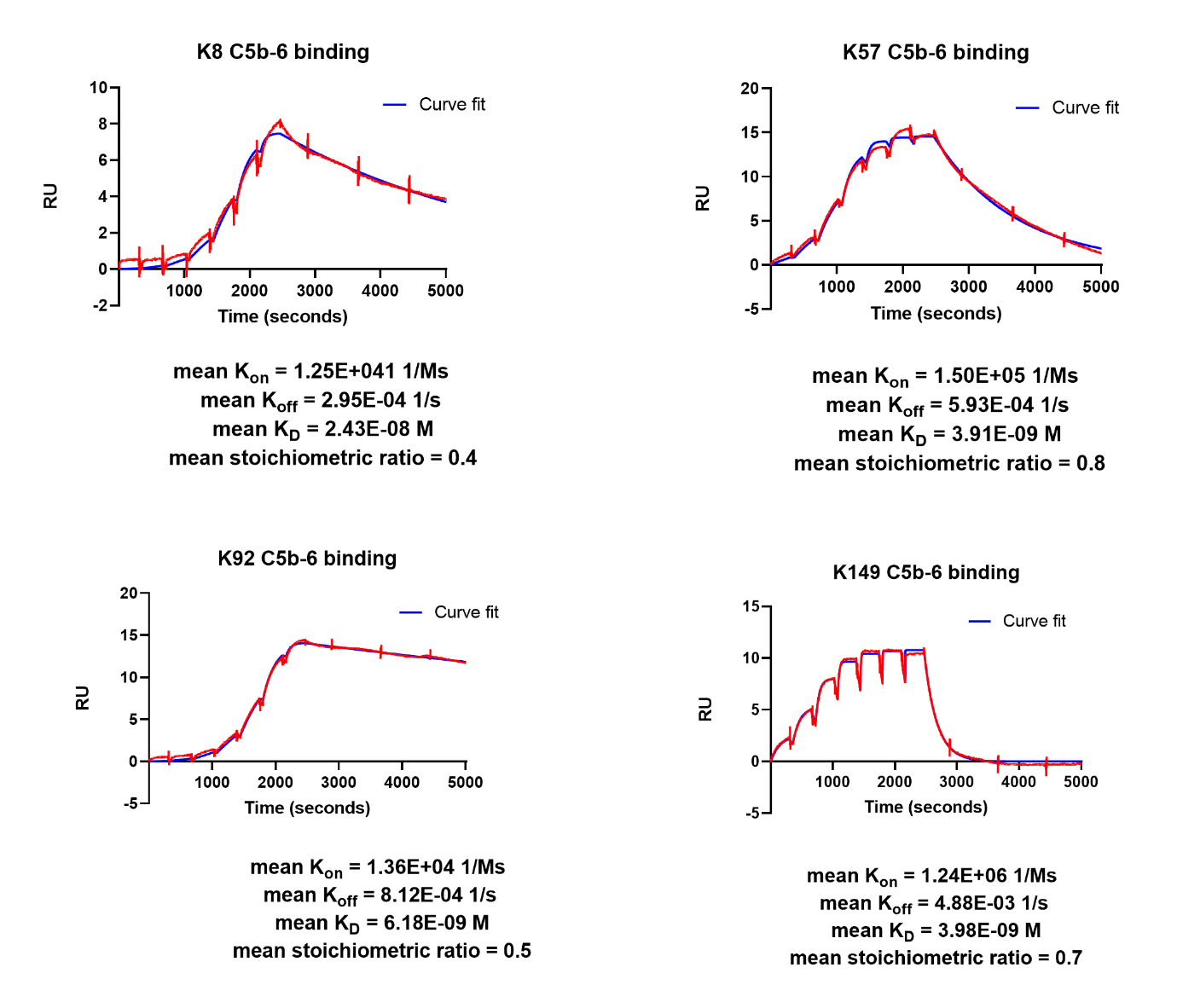

Supplement: Supplementary file 1. [file elife-63586-supp1.docx]
